# A naturally occurring carotenoid, lutein, reduces PDGF and H_2_O_2 _signaling and compromised migration in cultured vascular smooth muscle cells

**DOI:** 10.1186/1423-0127-19-18

**Published:** 2012-02-08

**Authors:** Huey-Ming Lo, Yih-Jeng Tsai, Wen-Yuan Du, Chih-Jen Tsou, Wen-Bin Wu

**Affiliations:** 1 Section of Cardiology, Department of Internal Medicine, Shin Kong Wu Ho-Su Memorial Hospital, Taipei, Taiwan; 2 Department of Otolaryngology, Shin Kong Wu Ho-Su Memorial Hospital, Taipei, Taiwan; 3 School of Medicine, Fu-Jen Catholic University, Taipei, Taiwan

**Keywords:** binding, carotenoid, lutein, migration, oxidative stress, signaling

## Abstract

**Background:**

Platelet-derived growth factor (PDGF) is a potent stimulator of growth and motility of vascular smooth muscle cells (VSMCs). Abnormalities of PDGF/PDGF receptor (PDGFR) are thought to contribute to vascular diseases and malignancy. We previously showed that a carotenoid, lycopene, can directly bind to PDGF and affect its related functions in VSMCs. In this study we examined the effect of the other naturally occurring carotenoid, lutein, on PDGF signaling and migration in VSMCs.

**Methods:**

Western blotting was performed to examine PDGF and H_2_O_2 _signaling. Flowcytometry was used to determine PDGF binding to VSMCs. Fluorescence microscopy was performed to examine intracellular ROS production. Modified Boyden chamber system (Transwell apparatus) was used for migration assay.

**Results:**

Lutein reduced PDGF signaling, including phosphorylation of PDGFR-β and its downstream protein kinases/enzymes such as phospholipase C-γ, Akt, and mitogen-activated protein kinases (MAPKs). Although lutein possesses a similar structure to lycopene, it was striking that lutein inhibited PDGF signaling through a different way from lycopene in VSMCs. Unlike lycopene, lutein not only interacted with (bound to) PDGF but also interfered with cellular components. This was evidenced that preincubation of PDGF with lutein and treatment of VSMCs with lutein followed by removing of lutein compromised PDGF-induced signaling. Lutein reduced PDGF-induced intracellular reactive oxygen species (ROS) production and attenuated ROS- (H_2_O_2_-) induced ERK1/2 and p38 MAPK activation. A further analysis indicated lutein could inhibit a higher concentration of H_2_O_2_-induced PDGFR signaling, which is known to act through an oxidative inhibition of protein tyrosine phosphatase. Finally, we showed that lutein functionally inhibited PDGF-induced VSMC migration, whereas its stereo-isomer zeaxanthin did not, revealing a special action of lutein on VSMCs.

**Conclusions:**

Our study reveals a differential action mechanism of lutein from other reported caroteinoids and suggests a possible beneficial effect of lutein but not zeaxanthin on prevention of vascular diseases.

## Background

Abnormal vascular smooth muscle cell (VSMC) proliferation and migration play an important role in the development and progression of proliferative cardiovascular diseases (CVDs), including hypertension, restenosis, and atherosclerosis [1-3].

Platelet-derived growth factor (PDGF) is a potent stimulator of growth and motility of connective tissue cells such as fibroblasts and SMCs [4]. PDGF is a dimeric molecule consisting of disulfide-bonded A and B-polypeptide chains. Homodimeric (PDGF-AA, PDGF-BB) as well as heterodimeric (PDGF-AB) isoforms exert their effects on target cells by binding with different specificities to two structurally related protein tyrosine kinase receptors, denoted α- and β-receptors [4,5]. Abnormalities of PDGF receptor (PDGFR)/PDGF are thought to contribute to a number of human diseases, including malignancy and vascular diseases.

PDGF participates in stimulating SMC proliferation and migration during atherosclerosis [6]. Expression of PDGF is low in normal blood vessels, but the levels of PDGF mRNA are increased following vascular smooth muscle cell transition into a synthetic state in culture [7] or after injury *in vivo *[8]. PDGF and its cognate receptors are also expressed in tumors [9]. PDGF stimulates autocrine growth of tumor cells and regulate tumor stromal fibroblasts and tumor angiogenesis [10]. Overexpression of PDGF receptor and/or ligand is found in brain tumors and diverse malignancies.

In addition to PDGF, vascular injury also induces oxidative stress and elevated production of reactive oxygen species (ROS) in the vessel wall [11,12]. Oxidative stress has been suggested to play an important role in the pathogenesis of CVDs, mainly through oxidative modification of low density lipoprotein, which initiates vascular inflammation and atherosclerotic lesion formation [13]. The most important ROS for pathological conditions are superoxide (O^2-^) and hydrogen peroxide (H_2_O_2_). Inhibition of ROS reduces vessel remodeling and restenosis [14]. Moreover, PDGFR activation increases intracellular ROS production and mediates PDGF signal transduction [15]. It was reported that both PDGF and extracellular H_2_O_2 _at a higher concentration stimulation lead to intracellular ROS production and regulate protein tyrosine phosphatase (PTP), which induces an elevation of tyrosine-phosphorylated proteins [16-18].

Lutein and its stereo-isomer, zeaxanthin, are carotenoids without provitamin A activity and found in a wide variety of fruits and vegetables, including cooked spinach, lettuce, broccoli, peas, lima beans, orange juice, celery, string beans, and squash [19,20]. It has been reported that higher quantities of dietary lutein were associated with lower risks of total stroke in the Health Professionals' Follow-Up Study [21]. Moreover, two other key studies have provided support for a role of lutein and zeaxanthin in prevention of cardiovascular diseases, which shows inverse correlation of plasma lutein concentration and carotid intima-media thickness [20]. In an *in-vitro *study, lutein and other carotenoids such as lycopene have been shown to reduce adhesion molecules expression in human aortic endothelial cells [22]. This reflects a possible role of lutein in the prevention of atherosclerosis. Lutein exists in high concentration in the macula [23]. However, dietary lutein stimulated delayed type hypersensitivity response, the number of CD4+ Th cells, and IgG production in dogs [24], suggesting its presence in peripheral areas and a possible protective role of lutein in vascular system.

We previously demonstrated that lycopene inhibits VSMC proliferation and migration through direct interaction with PDGF [25,26]. The predominant carotenoids found in human plasma are lycopene, β-carotene, and lutein, and their concentrations vary from 0 to 8 μM depending upon dietary intake [27]. In this study we evaluated lutein and its stereo isomer zeaxanthin on VSMC migration and PDGF signaling. Our results revealed a differential action mechanism of lutein from lycopene in inhibiting PDGF signaling and an opposite action of lutein and zeaxanthin on VSMC migration.

## Materials and methods

### Materials

The inhibitors for mitogen activated protein kinases (MAPKs) and phosphoinositide-3-kinase (PI-3K), bovine type I collagen and (+/-)-6-hydroxy-2,5,7,8-tetramethylchromane-2-carboxylic acid (Trolox) were purchased from Sigma Chemical Co. (St Louis, MO, USA). Hydrogen peroxide (H_2_O_2_) was from Merck KGaA Co. (Darmstadt, Germany). Antibodies (Abs) raised against phospho-ERK1/2 and PDGFR-β were from Santa Cruz Biotechnology (Santa Cruz, CA, USA). Ab raised against phospho-PDGFR was from Upstate Biotech Inc. (Lake Placid, NY, USA). Abs directed against PLCγ1, phospho-PLCγ1 (Tyr^783^), total p38 MAPK, phospho-p38 MAPK and total Akt were from Cell Signaling Technology, Inc. (Danvers, MA, USA). Recombinant PDGF-BB and Ab for total ERK1/2 were from R&D systems, Inc. (MN, USA). Lutein and zeaxanthin were purchased from Extrasynthese (Genay cedex, France) and were dissolved in dimethyl sulfoxide (DMSO) and tetrahydrofuran, respectively.

### Cell Culture

The animal experimental procedures were approved by the Fu-Jen Animal Experiment Committee. Rat aortic SMCs were isolated and characterized as previously described [25] and cultured in Dulbecco's modified Eagle's medium (DMEM) containing 10% fetal bovine serum (FBS), penicillin (100 units/ml), streptomycin (100 μg/ml) and fungizone (250 ng/ml) (Invitrogen Life Technologies, Carlsbad, CA, USA). Four to six passage cells were used in this study. Unless otherwise indicated, cells reaching 80-90% of confluency were starved and synchronized in DMEM at 37°C for 24 h and then subjected to further analysis.

### Cell lysate preparation and Western blot analysis

Cell lysate was prepared as previously described [26]. Total proteins were separated by electrophoresis on SDS-polyacrylamide gels, electroblotted onto PVDF membranes, and then probed using a primary mAb. Immunoblots were detected by enhanced chemiluminescence reagent (Perkin-Elmer, Waltham, MA, USA). For some experiments, membranes were stripped with a striping buffer (62.5 mM Tris-HCl, pH 6.7, 2% SDS and 100 mM β-mercaptoethanol), washed, and reprobed with Abs for the levels of α-tubulin or the corresponding total proteins and developed as described above.

### Cell migration assay

Migration assay with VSMCs was performed using a modified Boyden chamber model (Transwell apparatus, 8.0-μm pore size, Costar). Briefly, the lower face of polycarbonate filter (Transwell insert) was coated with type I collagen (10 μg/ml) for 30 min in the laminar flow hood. The lower chamber was filled with serum-free, PDGF-BB-containing medium preincubated with vehicle (DMSO) or lutein for 30 min. VSMCs (2.5 × 10^5 ^cells/ml) were plated to the upper chamber in the presence of vehicle or lycopene. After 3 h of incubation, all nonmigrant cells were removed from the upper face of the Transwell membrane with a cotton swab and migrant cells were fixed and stained with 0.5% toluidene blue in 4% paraformaldehyde. Migration was quantified by counting the number of stained cells per × 100 field (high power field, HPF) under the phase-contrast microscope (Leica DMIL^®^) and photographed.

### Flowcytometric analysis of PDGF-BB binding to VSMCs

Flowcytometric analysis of PDGF binding to VSMCs was performed according to the manufacturer's protocol of the PDGF-BB biotinylated fluorokine kit (R&D systems, Inc., MN, USA). Briefly, cells were incubated at 37°C for 1 h to allow regeneration of the receptors. Cells (1 × 10^5^) were then stained with biotinylated protein (soybean trypsin inhibitor, as a negative control) or biotinylated PDGF-BB at 4°C for 1 h in the presence of lutein or anti-PDGF-BB blocking Ab. After incubated with the fluorescein-conjugated avidin, cells were analyzed immediately by Partec CyFlow ML cytometer (Partech GmBH, Munster, Germany) using excitation and emission wavelength at 488 and 525 nm, respectively. Fluorescence signals from 7,500 cells were collected to calculate mean fluorescence intensity of a single cell.

### Fluorescence microscopic analysis of intracellular ROS level

Intracellular production of ROS was determined by the fluorescence microscopy as previously described [28] with minor modifications. Briefly, VSMCs were pretreated with lutein (10 μM) for 30 min and followed by stimulation with vehicle- or lutein-pretreated PDGF (10 ng/ml) at 37°C for 10 min. After a brief wash, cells were loaded with CM-H_2_DCFDA (5 μg/ml, Invitrogen) and incubated at 37°C for 15 min. After washed with PBS, cells were immediately analyzed under the fluorescence microscope (Nikon Eclipse Ti-S, Japan) using excitation and emission wavelength at 485 and 525 nm, respectively, and photographed by a digital camera. The fluorescence intensity of the positive staining cells was calculated based on their gray levels (from 0 to 255), as judged by image analysis software of Image-Pro Plus (Media Cybernetics, Inc., Baltimore, MD, USA).

### Statistical analysis

Data were expressed as mean ± standard error mean (SEM). Comparison of means of two groups of data was made by using the unpaired, two-tailed Student *t *test.

## Results

### Lutein inhibits PDGF signaling in VSMCs

**Figure 1:**
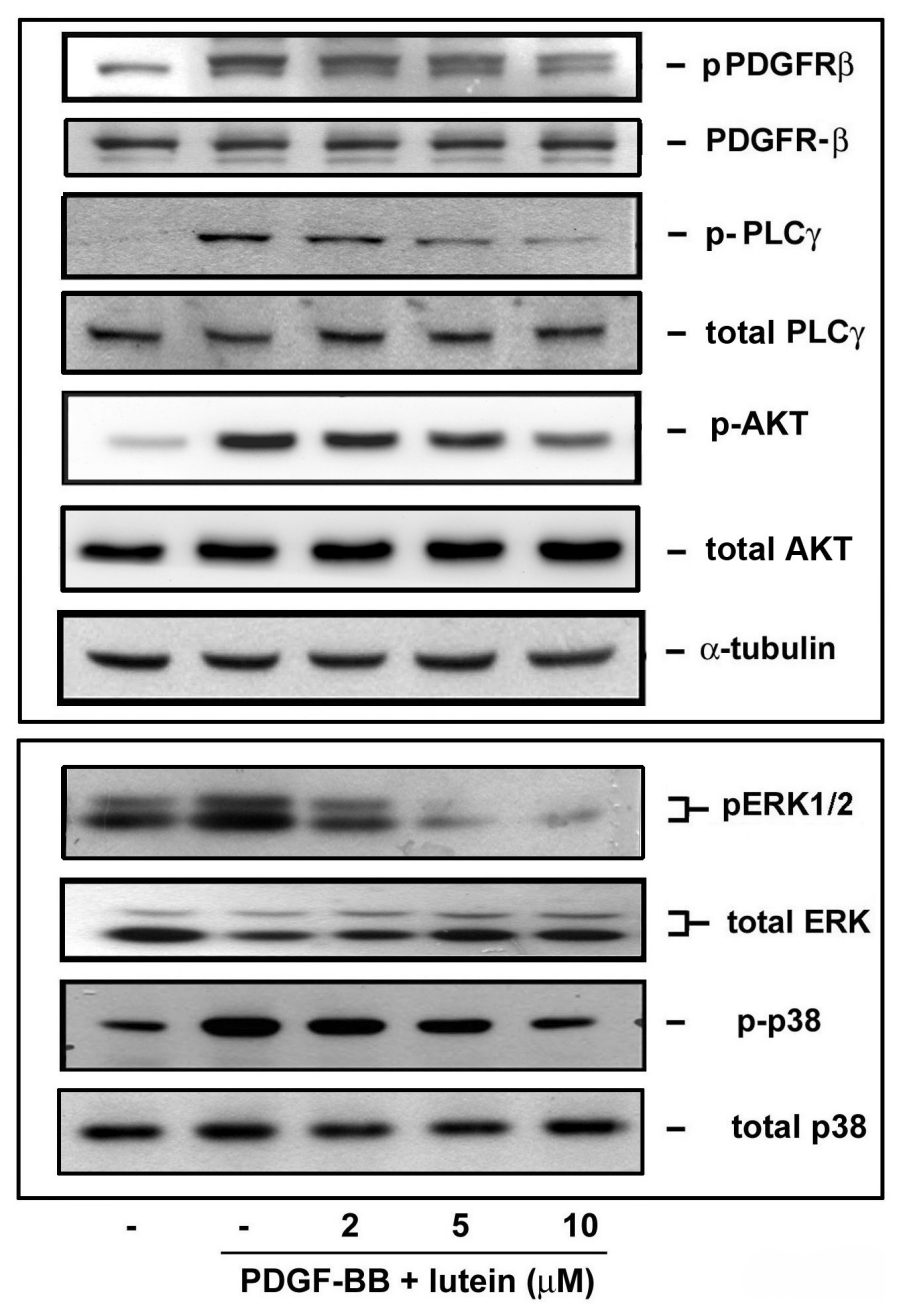
**Dose-dependent effect of lutein on PDGF-induced cellular signaling**. PDGF-BB was preincubated with vehicle or the indicated concentrations of lutein for 30 min and was added to VSMCs for 10 min. Cells were harvested and lysates were analyzed by Western blotting (n = 4).

**Figure 2:**
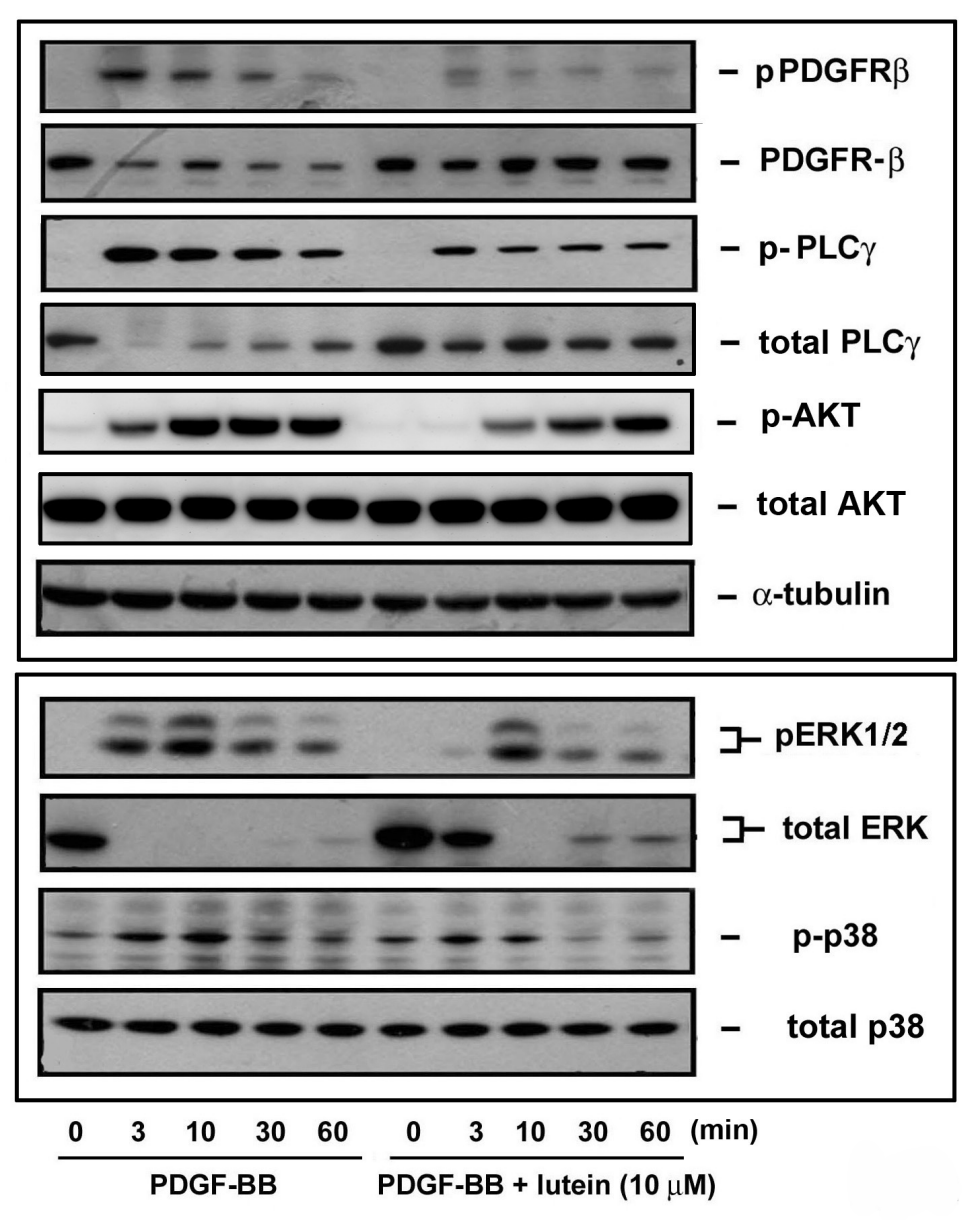
**Time-dependent effect of lutein on PDGF-induced cellular signaling**. PDGF-BB was preincubated with vehicle or lutein for 30 min and was added to VSMCs for the indicated time periods. After incubation, cells were harvested and lysates were analyzed by Western blotting (n = 3).

It has been reported that PDGF-BB binding to PDGFR (PDGF receptor) is associated with activation of PDGFR-tyrosine kinase activity [4], subsequently causing VSMC proliferation and migration through activation of downstream signaling [29]. To examine the effect of lutein on PDGF-BB-induced signal transduction pathway, the activation of PDGFR-β and its downstream kinases in VSMCs was determined. It was found that lutein inhibited PDGF-BB-induced PDGFR-β, PLCγ, Akt, and MAPKs phosphorylation in a concentration- and time-dependent manner (Figures 1 and 2).

### Lutein affects PDGF signaling through interaction with both PDGF and cellular components

**Figure 3:**
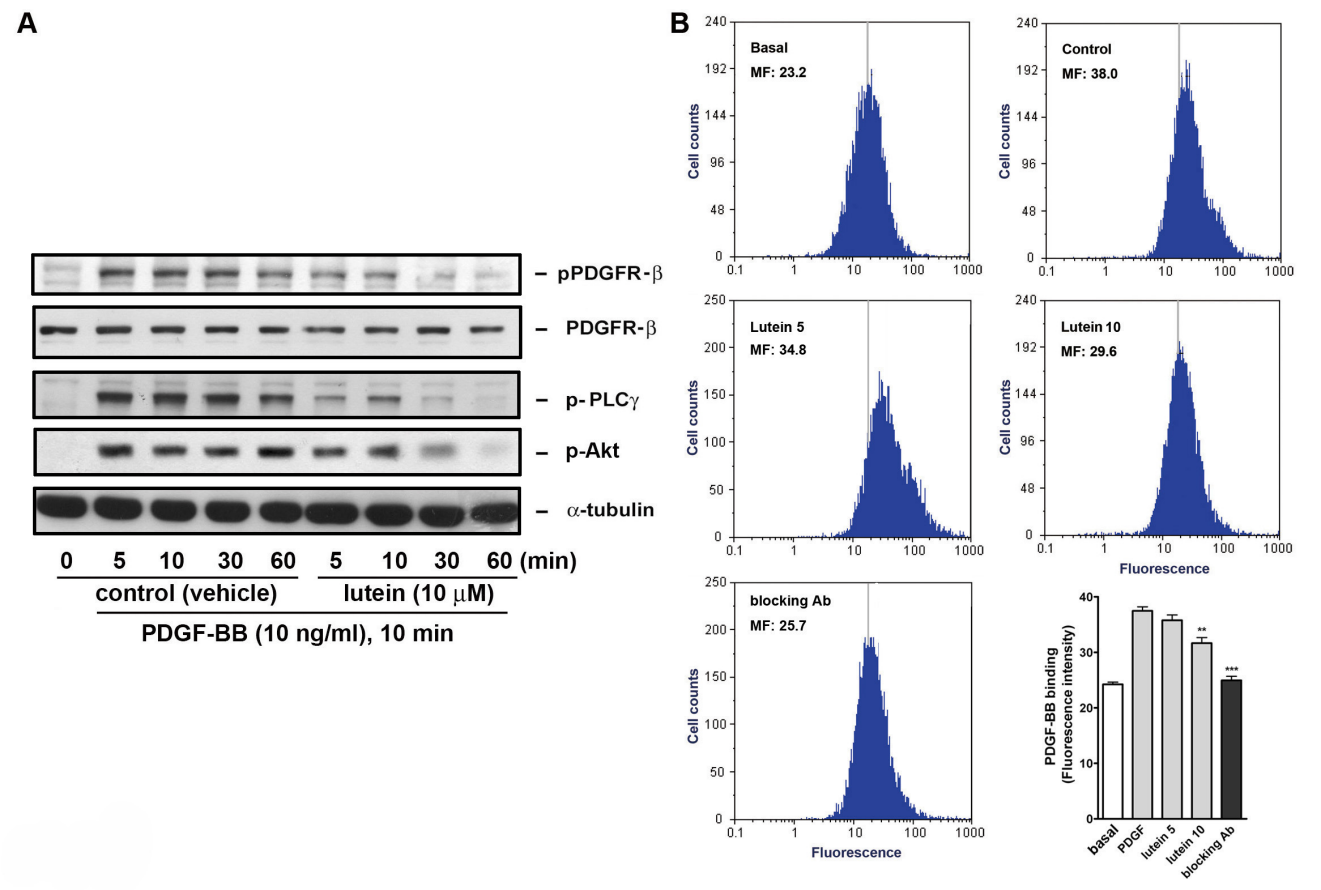
**Lutein interferes with PDGF binding to VSMCs**. (A) PDGF was preincubated with vehicle (control) or lutein for the indicated time and was added to stimulate cells for 10 min. Cells were then harvested and lysates were analyzed by Western blotting (n = 3). (B) Flowcytometric analysis of PDGF binding to VSMCs. Fluorescein-labeled soybean trypsin inhibitor (basal) or PDGF were added to VSMCs in the presence of DMSO (control), lutein (5 and 10 μM), or anti-PDGF blocking Ab at 4°C for 1 h, cells were analyzed by flowcytometry. The representative histograms and quantitative data were shown (n = 3-4).

**Figure 4:**
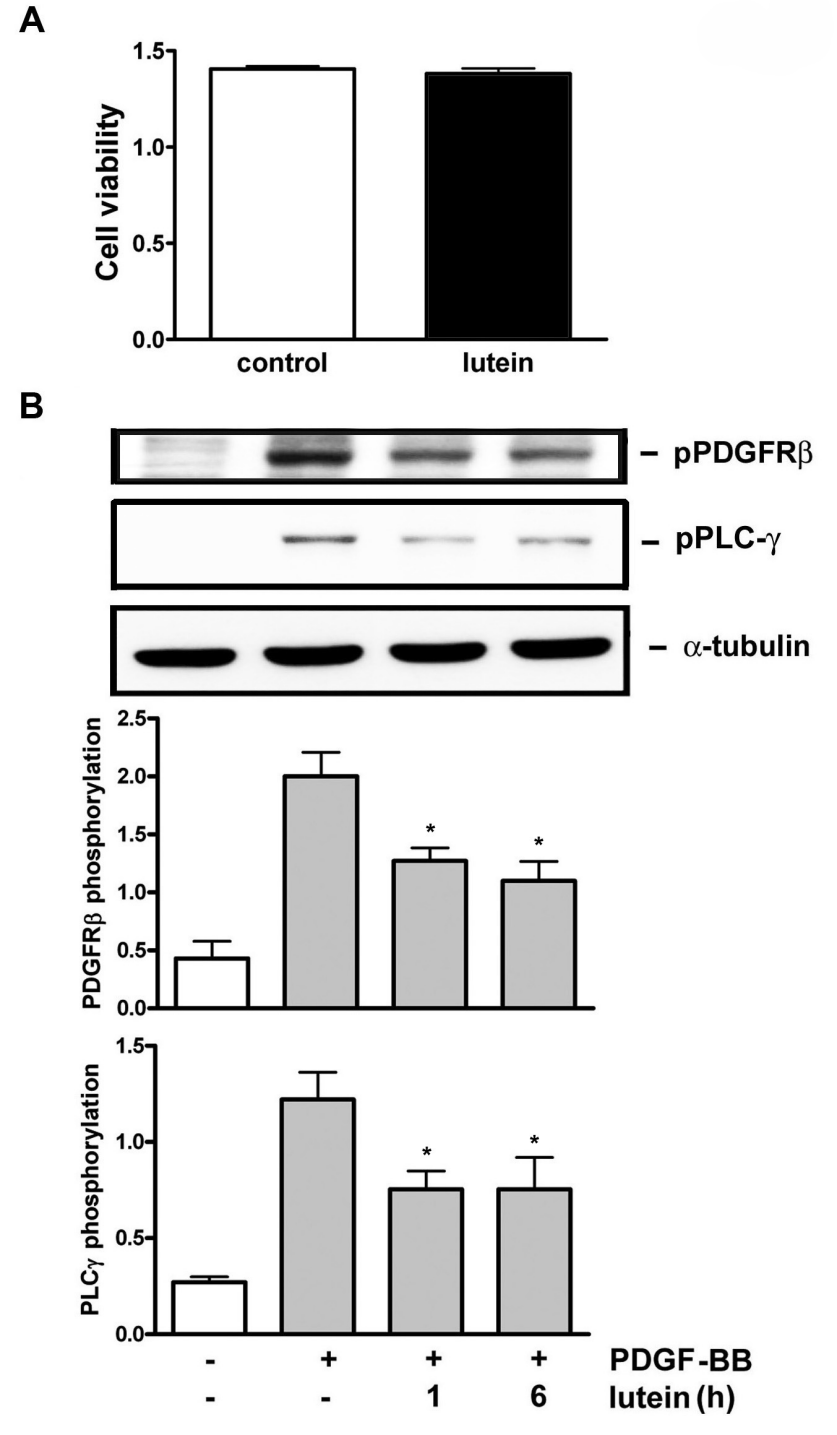
**Lutein affects cellular components**. (A) Cells were incubated with vehicle or lutein (10 μM) for 16 h and cell viability was measured by MTT assay as described in Methods. (B) Cells were preincubated with vehicle or lutein (10 μM) for 1 or 6 h and followed by an extensive wash to remove extracellular unbound lutein. Cells were then stimulated with PBS or PDGF for 10 min. PDGFR and PLCγ phosphorylation was examined by Western blotting and quantitation was performed by densitometry (n = 3).

In our previous study we have demonstrated that PDGF signaling affected lycopene is not through intracellular inhibition but is through direct interaction with PDGF [25,26]. Because lutein reduced PDGF signaling (Figures 1 and 2), we examined whether lutein acted similarly to lycopene. First, the effect of lutein on PDGF was examined using the pharmacological indirect approach. Medium containing PDGF-BB was preincubated with vehicle or lutein for a certain time and was added to VSMCs. The preincubation resulted in a time-dependent inhibition on PDGF-induced PDGFR-β, PLC-γ, and Akt phosphorylation, while PDGF signaling was not affected in vehicle-treated control cells. Moreover, the inhibition was pronounced at 30- and 60-min preincubation of PDGF with lutein (Figure 3A), suggesting that lutein directly interacted with PDGF. To confirm lutein interacting with PDGF, we performed PDGF binding assay. As determined by flowcytometric analysis, the mean fluorescence of VSMCs was increased by adding fluorescein-labeled PDGF but was decreased in the presence of an anti-PDGF blocking Ab, suggesting a specific interaction between PDGF and VSMCs. Lutein slightly inhibited PDGF binding at 5 μM but markedly inhibited PDGF binding to VSMC at 10 μM (Figure 3B). Secondly, we determined whether lutein affected cellular components during PDGF signaling. To ensure lutein entering into cytosol, cells were preincubated with lutein for a longer time (1 and 6 h) and followed by an extensive wash to remove extracellular unbound lutein. Cells with a slight orange color suggest an uptake of lutein into intracellular space (data not shown). Under this condition, cell viability was not significantly altered (Figure 4A). However, PDGF-BB-induced PDGFR-β and PLC-γ phosphorylation was affected in VSMCs pretreated with lutein (Figure 4B), indicating an inhibition on intracellular components by lutein.

### Lutein inhibits PDGF-induced ROS production and oxidative stress-induced signaling in VSMCs

**Figure 5:**
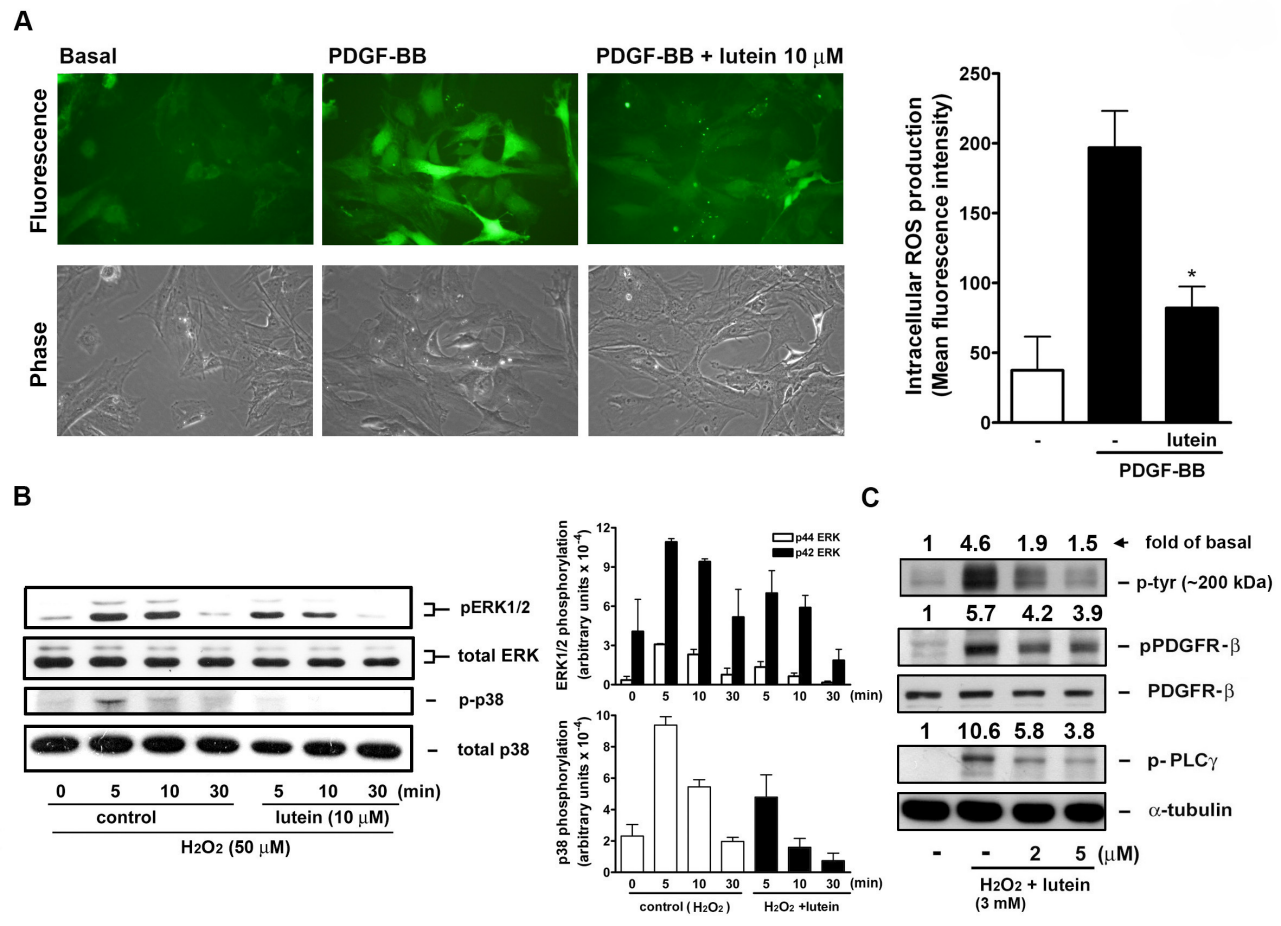
**Effect of lutein on PDGF-induced intracellular ROS production and H_2_O_2 _signaling**. (A) VSMCs were treated with PDGF-BB (PDGF, 10 ng/ml) in the presence of vehicle (veh) or lutein (10 μM) for 10 min. Cells were immediately analyzed by fluorescence microscopy. The representative data from an experiment were shown (n = 5). The mean fluorescence intensity from the positive staining cells was calculated by the image analysis software. *P < 0.05 vs. control. (B and C) VSMCs were pretreated with vehicle (veh) or lutein for 30 min and followed by the addition of (B) H_2_O_2 _(50 μM) or (C) H_2_O_2 _(3 mM) for the indicated time. Cellular signaling was determined by Western blotting (n = 3). Quantitation of MAPKs and PDGFR and PLCγ phosphorylation was performed by densitometry. Data were expressed as (B) a bar graph or (C) indicated by a number.

Since intracellular components were affected by lutein during PDGF signaling. Next, we tested whether lutein could affect PDGF-induced intracellular ROS production. As shown in Figure 5A, an obvious increase in intracellular ROS production was observed after VSMCs stimulated with PDGF-BB. Lutein markedly attenuated PDGF-induced intracellular ROS production, i.e. a decrease in fluorescence-positive cells. Further, we investigated the effect of lutein on H_2_O_2_-induced signaling in VSMCs. As reported by others, in our experimental system 50 μM of H_2_O_2 _could activate MAPKs signaling, including activation of ERK1/2 and p38 MAPK within 30-min stimulation. The activation by H_2_O_2 _was inhibited by lutein, whereas total MAPKs were not affected (Figure 5B). Since H_2_O_2 _at a higher concentration can directly activate PDGFR-β and its downstream signaling components in VSMCs through redox inactivation of PTP [30], we also examined whether lutein affected H_2_O_2 _(3 mM)-induced PDGF signaling. It was found that H_2_O_2_-induced PDGFR and PLC-γ phosphorylation was inhibited in a concentration-dependent manner.

### MAPKs, PI-3K, and ROS are involved in PDGF-BB-induced VSMC migration

**Figure 6:**
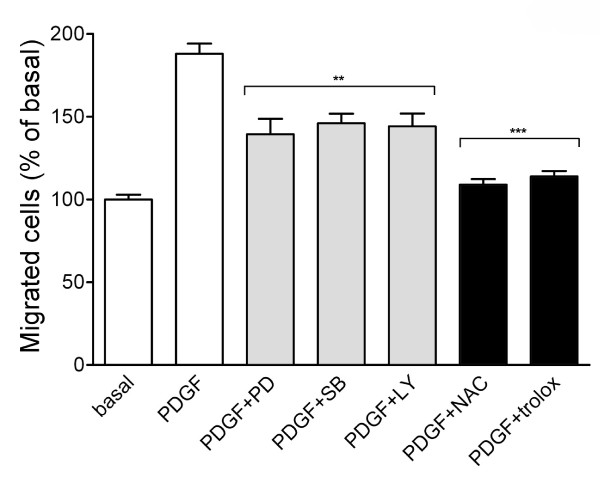
**Effect of kinase and ROS inhibitors on PDGF-BB-induced VSMC migration**. VSMCs were preincubated with DMSO, kinase inhibitors (10 μM; PD for PD98059, SB for SB202190, and LY for LY294002) or ROS inhibitors (NAC, 10 mM; trolox, 200 μM) for 30 min. Cells were added in the upper chamber and assembled with the lower chamber containing media in the presence of PBS (basal) or PDGF-BB (10 ng/ml). After incubation for 3 h, cells migrated to the underside of filter membrane were photographed as described in Methods. Cell migration was expressed as percentage of basal. **P < 0.01 vs. control, ***P < 0.001 vs. control.

It has been reported that PDGF causes cell proliferation and migration through activation of ERK1/2 and other signaling enzymes [4,29]. Moreover, antioxidants have been shown to inhibit PDGF-induced VSMC migration [17]. However, the PDGFR downstream signaling components responsible for VSMC migration have not been demonstrated in our system. Therefore, we examined the role of MAPKs, PI-3K, and ROS in VSMC migration. The following inhibitors were used in the migration assay, including PD98059 (an inhibitor targeting ERK signaling), SB202190 (a p38 MAPK inhibitor), LY294002 (a PI-3K inhibitor), N-acetylcysteine (NAC) and Trolox (a vitamin E derivative). Figure 6 showed that some VSMC migration toward collagen in a basal condition. By PDGF-BB stimulation, VSMC migration was apparently increased about 2 folds. It was found that PDGF-BB-induced VSMC migration was attenuated by PD98059, SB202190 and LY294002 but was markedly inhibited by the ROS inhibitors (Figure 6). The inhibitory profile was different from those on PDGF-AA-induced migration in VSMCs [26], excluding the possibility of cytotoxicity exhibited by these inhibitors. Taken together, the result revealed that activation of ERK, p38 MAPK, PI-3K and ROS was essential for PDGF-BB-induced migration.

### Lutein but not zeaxanthin inhibits PDGF-BB-induced VSMC migration

**Figure 7:**
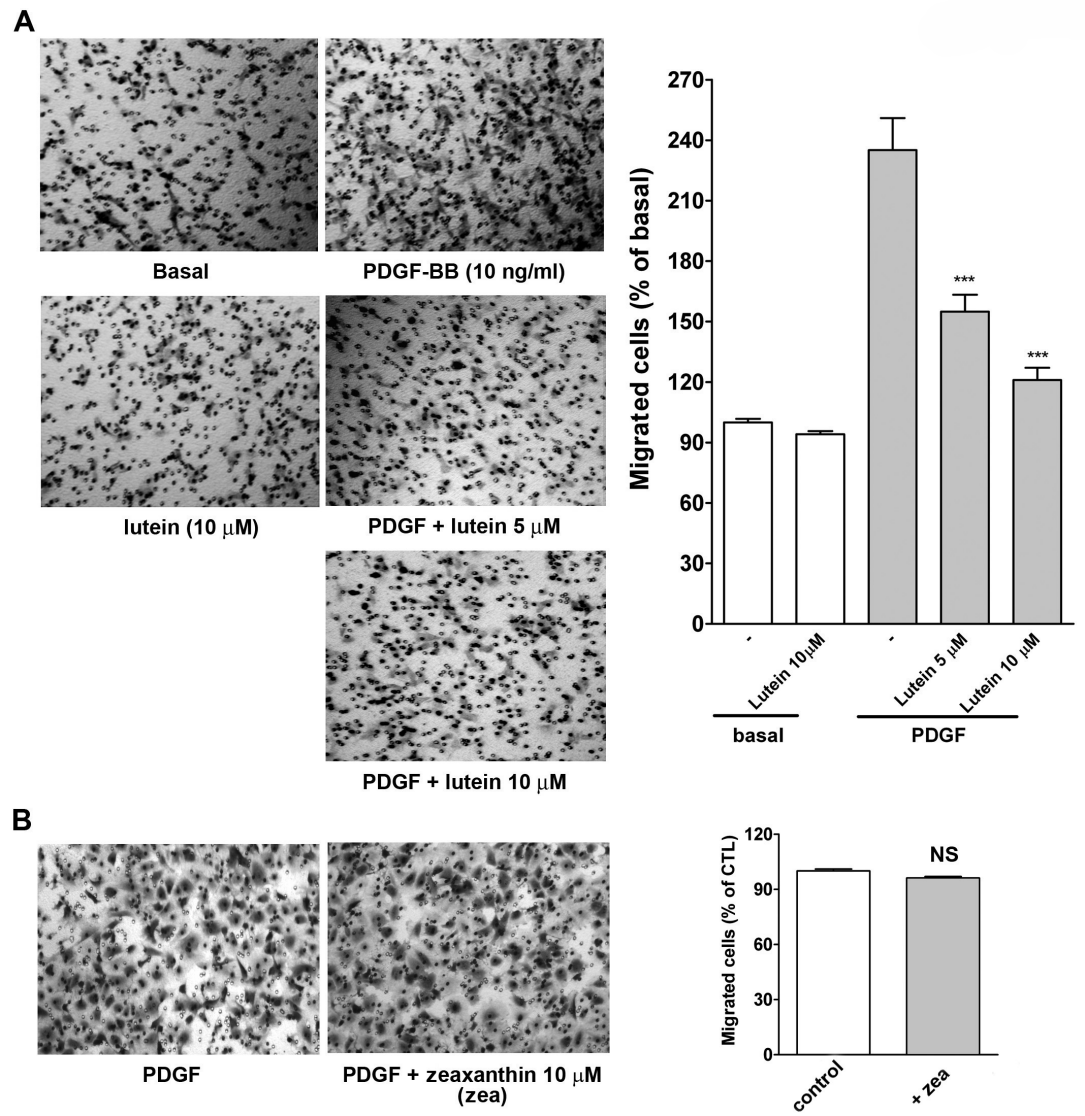
**Lutein but not zeaxanthin inhibits VSMC migration**. VSMCs were seeded in the upper chamber precoated with collagen. The inserts were assembled with the lower chamber, which was filled with serum-free (basal) or PDGF-containing medium preincubated with vehicle or (A) lutein or (B) zeaxanthin (zea) for 30 min. After incubation for 3 h at 37°C, fixation was performed and nonmigrated cells were removed. Cells migrated to the underside of filter membrane were photographed and counted in high-power field (magnification, 100 ×) under the microscope. Right panels: a quantitative analysis of migrated cells. All experiments were conducted in duplicate. Cell migration was expressed as percentage of basal or control (n = 6-8). ***P < 0.001 versus control. NS: non significance.

**Figure 8:**
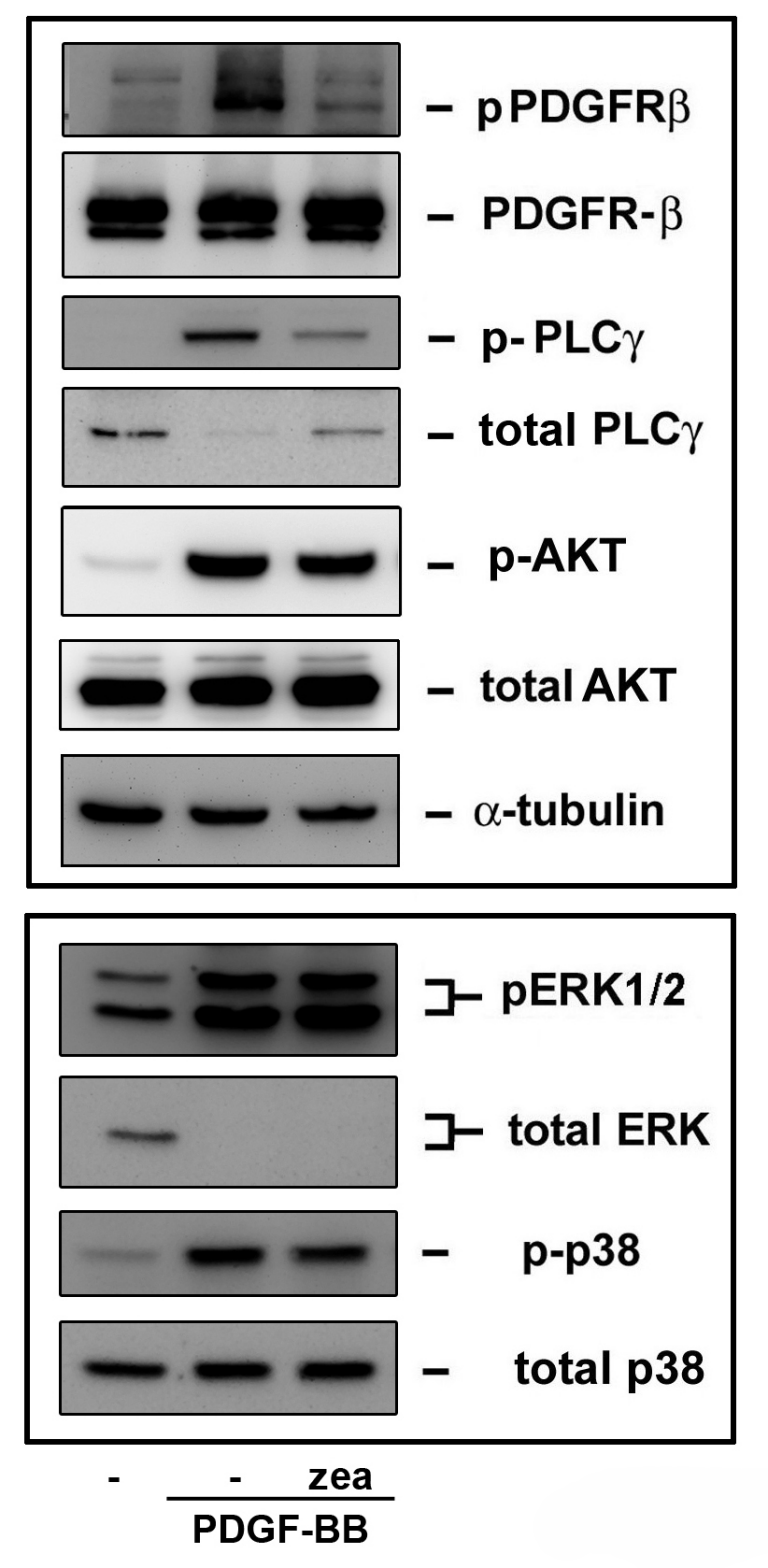
**Effect of zeaxanthin on PDGF-BB signaling**. PDGF-BB (10 ng/ml) was preincubated with vehicle or zeaxanthin (zea, 10 μM) for 30 min and was added to VSMCs for 10 min. Cells were harvested and lysates were analyzed by Western blotting (n = 3-4).

Lutein elicited an inhibitory effect on PDGF-BB-induced signal transduction pathway such as PDGFR, PLCγ, MAPKs, and ROS production. Next, we examined whether lutein functionally affected PDGF-BB-induced VSMC migration. As shown in Figure 7A, lutein did not inhibit basal migration, but it inhibited PDGF-BB-induced migration in a concentration-dependent manner in VSMCs. Interestingly, zeaxanthin, a stereo-isomer of lutein, did not significantly inhibit PDGF-BB-induced migration even at 10 μM (Figure 7B). However, a further analysis showed that zeaxanthin could inhibit PDGF-BB-induced PDGFR and PLCγ activation, but it only slightly affected Akt, ERK1/2, and p38 MAPK activation (Figure 8).

## Discussion

PDGF has been demonstrated as a critical growth factor in participating in the development of vascular diseases and tumor. Several natural compounds, such as (-)-epigallocatechin-3-gallate (a tea polyphenol) [31,32], luteolin (a flavonoid) [33] and chrysin [18], have been demonstrated to affect PDGF-induced signaling, migration, or proliferation. In our previous study lycopene was able to inhibit PDGF-AA, -AB and -BB-induced signaling and reduce balloon-induced neointima formation in rat carotid artery injury [25,26]. In this study, we presented findings to demonstrate that the carotenoid lutein inhibited PDGF-induced signaling and functionally blocked migration in VSMCs (Figures 1, 2, and 7). Moreover, lutein inhibited oxidative stress (H_2_O_2_)-induced signaling and a higher concentration of H_2_O_2_-induced signaling (Figure 5). In striking contrast, the isomer zeaxanthin did not affect VSMC migration even at an equal concentration with lutein (Figure 7). Therefore, our results suggest that these carotenoids act in a differential way in affecting PDGF signaling and functions in VSMCs.

Regarding how lutein affected cellular signaling in VSMCs, it was found that lutein reduced PDGF signaling in a time- and concentration-dependent manner (Figures 1 and 2). The effect was profound when PDGF was preincubated with lutein (Figure 3A), suggesting lutein interacted with PDGF and interfered with PDGF binding to its cognate receptors. This was confirmed by the flowcytometric analysis that fluorescein-labeled PDGF binding to VSMCs was significantly reduced by lutein at 10 μM (Figure 3B). However, unlike lycopene, lutein attenuated VSMCs signaling through affecting cellular components. When cells were preincubated with lutein and followed by an extensive wash to remove extracellular lutein that may interact with PDGF, PDGF signaling was also significantly reduced (Figure 4B). This was not due to cytotoxicity by lutein because this interference did not cause any decreases in cell viability (Figure 4A). It has been reported that PDGFR activation enhances intracellular reactive oxygen species (ROS) production and mediates PDGF signal transduction [15]. PDGF and extracellular H_2_O_2 _stimulation lead to intracellular ROS production and regulate protein tyrosine phosphatase (PTP), which induces an elevation of tyrosine-phosphorylated proteins [16]. In this study we observed an elevation of intracellular ROS production after PDGF stimulation by fluorescence microscopy. This increase was abrogated by lutein (Figure 5A), suggesting that lutein might act as a ROS scavenger or an inhibitor affecting upstream of ROS. A further analysis confirmed lutein acting as a ROS scavenger because it inhibited H_2_O_2 _(50 μM)-induced ERK1/2 and p38 MAPK activation in VSMCs (Figure 5B), which was activated independent of PDGFR activation. This could be also demonstrated by the observation that lutein inhibited PDGFR signaling induced by a higher concentration of H_2_O_2 _(3 mM) (Figure 5C), which is known to directly activate PDGFR-β and its downstream signaling components in VSMCs through an intracellular ROS increase and redox inactivation of PTP [30]. Since PTP is responsible for dephosphorylating phosphorylated tyrosine residues in activated tyrosine kinases, this suggests a direct effect of lutein on ROS content or activated tyrosine kinases.

It is an interesting issue that the carotenoids with a similar structure act in a differential way on VSMCs. Our previous studies have shown that lycopene affects PDGF signaling through interaction with PDGF but not cellular components [25,26]. However, in this study we found that lutein affected PDGF binding, cellular components, and then migration, whereas zeaxanthin did not inhibit PDGF-induced migration (Figure 7B). The ineffectiveness of zeaxanthin on PDGF-induced migration is very intriguing. There is a report that zeaxanthin can inhibit PDGF-BB-induced migration in human dermal fibroblasts. The authors concluded that zeaxanthin affects cellular components but does not directly interact with PDGF-BB [34]. In our system, zeaxanthin was found to inhibit PDGF-BB-induced PDGFR and PLCγ activation; however surprisingly it only marginally affected Akt, ERK1/2, and p38 MAPK activation (Figure 8). Since ERK, p38 MAPK, PI-3K and ROS were required for PDGF-BB-induced VSMC migration (Figure 6), the less effectiveness of zeaxanthin on these kinases activation (Figure 8) may partly explain this phenomenon. Lycopene, lutein, and zeaxanthin are all isoprenoids with polyene skeleton (chain). Only lycopene is linear (acyclic) and the skeleton of the other two are modified by cyclization at both ends to give different end groups [35]. It has been reported that carotenoids are associated with their radical scavenging properties and their exceptional singlet oxygen quenching abilities [36]. However, some recent experiments by the authors using cells in culture have shown not only loss of antioxidant effectiveness but also pro-oxidant effects of carotenoids at high carotenoid concentrations [37-40]. This suggests a diverse effect of these carotenoids. Therefore, more efforts are needed to clarify whether the structural differences between these caroteinoids contribute to their differential cellular effects.

## Conclusions

In this study we provided evidence that lutein interacts with PDGF and affects cellular components, leading to interference with PDGF intracellular signaling, and functionally inhibits VSMC migration. We also demonstrated that zeaxanthin, the stereo-isomer of lutein, shows a distinct effect on PDGF signaling and VSMC migration. Our findings, together with our previous study, not only suggest the possible beneficial effect of lutein in preventing cardiovascular diseases but also an intriguing phenomenon of these carotenoids in inhibiting of PDGF signaling, highlighting their differences in mechanism of action in affecting VSMC behaviors.

## List of abbreviations used

CVD: Cardiovascular disease; ERK: extracellular matrix-regulated kinase; PDGF: platelet-derived growth factor; PDGFR: PDGF receptor; PLC-γ: phospholipase C-γ; MAPK: mitogen-activated protein kinase; VSMC(s): vascular smooth muscle cell(s).

## Competing interests

The authors declare that they have no competing interests.

## Authors' contributions

HML and WBW conceived of the study and designed research. HML, YJT, WYD and CJT performed research and analyzed data. WBW wrote the paper. All authors read and approved the final manuscript.
